# A survey to identify barriers of implementing an antibiotic checklist

**DOI:** 10.1007/s10096-015-2569-5

**Published:** 2016-01-25

**Authors:** F. V. van Daalen, S. E. Geerlings, J. M. Prins, M. E. J. L. Hulscher

**Affiliations:** Department of Internal Medicine, Division of Infectious Diseases, Centre for Infection and Immunity Amsterdam, Academic Medical Centre, Room F4-106, Meibergdreef 9, 1105 AZ Amsterdam, The Netherlands; Scientific Institute for Quality of Healthcare, Radboud University Medical Centre, Nijmegen, The Netherlands

## Abstract

A checklist is an effective implementation tool, but addressing barriers that might impact on the effectiveness of its use is crucial. In this paper, we explore barriers to the uptake of an antibiotic checklist that aims to improve antibiotic use in daily hospital care. We performed an online questionnaire survey among medical specialists and residents with various professional backgrounds from nine Dutch hospitals. The questionnaire consisted of 23 statements on anticipated barriers hindering the uptake of the checklist. Furthermore, it gave the possibility to add comments. We included 219 completed questionnaires (122 medical specialists and 97 residents) in our descriptive analysis. The top six anticipated barriers included: (1) lack of expectation of improvement of antibiotic use, (2) lack of expected patients’ satisfaction by checklist use, (3) lack of feasibility of the checklist, (4) negative previous experiences with other checklists, (5) the complexity of the antibiotic checklist and (6) lack of nurses’ expectation of checklist use. Remarkably, 553 comments were made, mostly (436) about the content of the checklist. These insights can be used to improve the specific content of the checklist and to develop an implementation strategy that addresses the identified barriers.

## Background

A better use of current antibiotic agents is necessary to help control antimicrobial resistance (AMR) [1]. Antibiotic stewardship programs (ASPs) are introduced to coordinate activities to measure and improve appropriate antibiotic use in daily hospital practice [2]. Recently, a set of generic quality indicators (QIs) was developed to measure appropriate antibiotic use in the treatment of bacterial infections in adult patients hospitalised at non-intensive care unit departments [3]. The next step is using these QIs to improve antibiotic use in daily practice. Checklists are effective tools to improve patient care [4–7]. For example, the use of a surgical safety checklist resulted in an absolute risk reduction in perioperative complications of 10.6 % [4], and a large and sustained reduction in rates of catheter-related bloodstream infections was demonstrated after the implementation of an infection control checklist [5]. It can, therefore, be hypothesised that the use of an antibiotic checklist consisting of generic quality indicators (Box 1) improves antibiotic use in the hospital. However, the implementation of a checklist needs to be combined with an understanding of barriers to its uptake, since it has been shown that physicians have resistances and interferences to the use of checklists [8, 9]. Without enough attention for such barriers that hinder implementation, the use of a checklist may fail, even where other studies showed striking improvements using the same checklist [9, 10]. Ideally, potential barriers hindering uptake are analysed before implementation, to influence both the type and content of the implementation activities [11]. In general, implementation can be complicated by barriers concerning the innovation itself, the individual professional, professional interactions, the patient, the organisation, incentives, resources or the socio-political context [12, 13]. Although addressing barriers that influence the effectiveness of an innovation to improve patient care is a crucial step in the knowledge-to-action process [14], very few barrier studies have been done prior to checklist implementation [15].

***Box 1: The antibiotic checklist based on generic quality indicators***

**Table:** 

1	Take at least two sets of blood cultures before starting systemic antibiotic therapy.
2	Take specimens for culture from suspected sites of infection, if possible before starting systemic antibiotic therapy, but at the latest after 24 hours of treatment.
3	Prescribe systemic antibiotic treatment according to the local antibiotic guideline.
4	a. Determine renal function.
	b. Adapt dose and dosing interval of systemic antibiotics to renal function if necessary.
5	Document the antibiotic treatment in the case notes or electronic medical record (EMR), including: - Indication; - Name; - Dose; - Interval; - Route of administration.
6	Determine whether antibiotic therapy can be adapted as soon as culture results become available.
7	Switch from intravenous to oral antibiotic therapy after 48–72 hours on the basis of the clinical condition, provided that oral treatment is adequate.^a^

^a^*Adequate means:*

*1: When the antibiotic is available orally;*

*2: When oral intake and gastrointestinal absorption are adequate;*

*3: Adequate in terms of diagnosis (exceptions are e.g. endocarditis, meningitis).*

Barrier studies performed during or after checklist implementation [16–24] described barriers such as lack of understanding the purpose of the checklist [17], duplication with current work [18, 19] and problems with the method of implementation [24].

The present study aims to identify barriers to the uptake of an antibiotic checklist in Dutch hospitals prior to checklist implementation and to select implementation activities to target the predominant barriers that obstruct checklist uptake.

## Methods

We performed an online questionnaire survey among medical specialists and residents to explore anticipated barriers hindering the uptake of an antibiotic checklist.

### Development of the antibiotic checklist barrier questionnaire

We based our questionnaire on the Dutch validated measurement instrument for determinants of innovations (MIDI), combined with barriers found in the literature. The MIDI is developed by the Netherlands Organisation for Applied Scientific Research (TNO) and is meant as a tool for researchers to survey determinants that influence the uptake of an innovation [13]. Additionally, we performed a literature search to find publications on barriers to checklist implementation and to appropriate antibiotic use. Box 2 shows the terms we used in our search, which resulted in 168 hits in total. We selected one book [25], three systematic reviews [12, 26, 27], eight relevant barrier studies [15–22, 24] and four studies on barriers to the appropriate use of antibiotics [28–31]. Based on this information, we adapted the MIDI to fit the topic of antibiotic use, so we removed potential barriers that were not relevant and added barriers that were mentioned in the literature.

***Box 2: Terms for literature search***

**Table:** 

Topic	Search terms in title	Hits
Reviews on barriers	(checklist* OR guideline*) AND (determinant* OR barrier* OR factor*) Filter: systematic reviews	130
Barriers to checklist implementation	(barrier* OR facilitator* OR determinant* OR challenge*) AND checklist*	13
Barriers to appropriate antibiotic use	(antibiotic* OR antimicrobial* OR antibacterial*) AND (barrier* OR behaviour* OR attitude*) AND (appropriate* [Title/Abstract] OR guideline* [Title/Abstract])	25

The final online questionnaire started with a description of the antibiotic checklist (Box 1) and was followed by 23 statements on anticipated barriers related to the checklist (seven items), the individual professional (six items), professional interactions (seven items), the patient (two items) and to resources (one item) (see Table 1 for the specific statements). To diminish the influence of the physician’s criticisms on the content of the checklist, statements 8 through 23 started with the sentence “Assuming that the checklist is adapted to your comments on its contents”. The level of agreement or disagreement with the statements was measured by a six-point Likert scale (1 = ‘totally agree’ and 6 = ‘totally disagree’). For each statement, it was possible to choose a seventh option: ‘I don’t know’.

**Table 1:** Survey questionnaire and results per domain

Domain	*N* ^a^	Yes, this is a barrier (%)	Top five
Checklist			
This checklist explains clearly what I have to do and in which order	219	3.7	
This checklist is based on evidence or experts’ consensus	192	7.8	
This checklist includes every step of appropriate antibiotic use in the hospital	216	15.3	
This checklist is too complex for use in daily practice	218	17.4	☒
This checklist fits in current practices	217	10.1	
The benefits of using the checklist are clear	217	13.4	
This checklist is feasible for all my patients who receive IV antibiotics	216	20.8	☒
Individual professional			
This checklist is a threat to my professional autonomy	216	13.4	
I expect that this checklist will improve the quality of my antibiotic prescriptions	212	26.9	☒
It is part of my job to use this checklist	215	16.7	
I am capable of using this checklist	211	5.2	
I have enough knowledge and expertise to use the checklist adequately	218	1.4	
I have good previous experiences with working with a checklist	201	19.9	☒
Professional interactions			
Colleagues will support me to use this checklist	183	8.7	
Supervisors will support me to use this checklist	177	6.8	
Nurses will support me to use this checklist	186	9.1	
Colleagues will use this checklist	191	14.7	
Colleagues will expect me to use the checklist	196	10.2	
Supervisors will expect me to use the checklist	179	9.5	
Nurses will expect me to use the checklist	182	14.8	
Patients			
Patient will be satisfied that this checklist is being used	170	21.8	☒
I expect that this checklist will improve the patient’s antibiotic treatment	212	12.3	
Resources			
There are enough financial resources to use the checklist as it is meant to be used	*108 of 219 (49.3 %) answered ‘I don’t know’ → exclusion*		

^a^ *N* Number of answers after exclusion of the answers ‘I don’t know’

Furthermore, physicians could criticise the separate components of the checklist by adding comments, and there was also space for general or organisational comments. The questionnaire was completed anonymously, but we asked for the participant’s function, department and hospital.

### Setting and participants

To gain insight into anticipated barriers to the uptake of an antibiotic checklist in hospitals prior to checklist implementation, physicians in nine Dutch hospitals were invited to participate in the survey. These nine hospitals, including two university and seven non-university hospitals, previously agreed to participate in a cluster-randomised trial on the implementation of the antibiotic checklist [32].

We visited the hospitals to inform the local antibiotic stewardship team about the antibiotic checklist and the questionnaire. Following this visit, we emailed the contact physician a link to the questionnaire, and he/she forwarded this email to the target group. The target group consisted of specialists and residents—with all levels of experience and various professional backgrounds—who have direct contact with and prescribe antibiotics to adult patients.

### Analysis

We included questionnaires in the analysis if at least half of the statements were appraised. We excluded statements from further analyses if ≥30 % of the participants answered ‘I don’t know’. While taking into account whether the statement was formulated as a barrier hindering uptake (“This checklist is a threat to my professional autonomy”) or as a facilitator helping uptake (“I expect that this checklist will improve the quality of my antibiotic prescriptions”), all answers (1 through 6) were re-coded into dichotomous scores: anticipated barrier ‘yes’ or ‘no’. The answers ‘I don’t know’ were excluded from the analyses. We computed frequencies and percentages and created a top five of the statements that were most often mentioned as barriers.

We categorised the comments on the checklist added by the participants. If comparable comments were mentioned three times or more, the comment was considered to be relevant. We also created a top five of comments.

## Results

### Participants

The online questionnaire was filled out by participants in eight of the nine hospitals that initially agreed to participate in the cluster-randomised trial on the implementation of the antibiotic checklist. One non-university hospital no longer wanted to participate and was replaced by a similar hospital. In another hospital, the link to the questionnaire was only emailed to physicians of the department of infectious diseases. In total, 250 physicians participated in the survey, of which 219 participants completed 50 % or more of the questionnaire statements. These 219 questionnaires were included in the analyses. The participants’ characteristics are summarised in Table 2. The number of completed questionnaires per hospital ranged from 8 to 90.

**Table 2:** Participants’ characteristics (*n* = 219)

	*n*
University/non-university	104/115
Specialists/residents	122/97
Specialties	
Internal medicine, gastroenterology and pulmonology	125
General surgery	27
Neurology	23
Emergency department	15
Urology	9
Gynaecology	5
Plastic surgery	3
Oral and maxillofacial surgery	3
Ear, nose and throat	2
Anaesthesia	2
Microbiology	2
Ophthalmology	1
Orthopaedic surgery	1

### Barriers

Table 1 shows the survey results. The statement concerning the availability of sufficient financial resources to use the checklist was excluded from further analysis, as more than 30 % of the participants answered ‘I don’t know’. The top five anticipated barriers were: (1) lack of expected quality improvement of the physician’s antibiotic prescribing (26.9 %), (2) lack of expected patients’ satisfaction with checklist use (21.8 %), (3) lack of feasibility of the checklist (20.8 %), (4) negative previous experiences with other checklists (19.9 %) and (5) the complexity of the antibiotic checklist (17.4 %).

To exclude the possibility that the single hospital in which 90 physicians completed the questionnaire influenced the results disproportionally, we compared the appraisals of the 219 participants (nine hospitals) with the appraisals of 129 participants (eight hospitals). The top five anticipated barriers from these eight hospitals differed on one statement: instead of ‘complexity of the checklist’, the statement ‘nurses will expect me to use the checklist’ was in the top five. For this reason, this barrier was added to the list of frequently mentioned barriers (6). This top six contains barriers from four different domains, namely the individual professional (1 and 4), the patient (2), the checklist (3 and 5) and professional interactions (6).

### Comments

In total 553, comments were given, of which 436 were comments and suggestions regarding the content of the checklist, 59 were general comments and 58 were organisational comments. These organisational comments described contextual factors that should be taken into account in these specific hospitals, i.e. implementation of a new electronic medical record (EMR) system (14.6 %) and merger of the hospital with another hospital (7.3 %). Comments on the content of the checklist or general comments that were relevant for all hospitals and were mentioned at least three times are presented in Table 3. The five most frequently mentioned comments were: (1c) “the item documentation leads to duplication of work” (11.0 %), (2c) “doubts about the need of blood cultures for several diagnoses” (10.5 %), (3c) “incomplete or too simplistic clarification of ‘adequate in terms of diagnosis’ for the item IV o oral switch” (8.2 %), (4c) “add information about the renal function” (6.8 %) and (5c) “add that one should take different sites for taking two different blood cultures” (6.4 %). Again, we compared the overall top five with the top five after exclusion of the 90 questionnaires of the single (university) hospital, which showed that the top five of the eight hospitals was equal to the overall top five comments.

**Table 3:** Comments per checklist item

Checklist item	Comment mentioned at least three times	*N* ^b^	Top five
Blood cultures (*n* = 88)^a^	Doubts about the need of blood cultures for several diagnoses (e.g. cellulitis)	23	☒
	Add in the checklist that one should take different sites for the two cultures	14	☒
	Logistically difficult because of lack of time	10	
	Doubts about the cost-effectiveness	10	
	Blood cultures should only be taken if the patient has fever	8	
	In which situation should you take more than two blood cultures?	8	
	Add in the checklist how long the period should be between the two cultures	6	
	This causes delay in the start of treatment in patients with a suspicion of bacterial meningitis	4	
	Make clear that one set exists of an aerobic and an anaerobic bottle	3	
Culture of suspected site of infection (*n* = 43)^a^	Only if possible	12	
	The mentioned timeframe for taking the culture (<24 h) causes confusion	9	
	This may delay the start of treatment	4	
	If there is no suspected site of infection, a urine culture is always indicated	3	
Prescribing antibiotics according to the hospital guidelines (*n* = 68)^a^	Add a link to the guidelines	10	
	It is not clear what is meant by hospital guidelines, e.g. some departments have their own protocols. Do these count as guidelines?	5	
	If previous culture results are available, these should be taken into account	4	
	Why not use national guidelines as a first choice?	3	
Adapt to renal function (*n* = 46)^a^	Provide the normal range for renal function, or provide criteria for adaptation, or add a link to these criteria	15	☒
	Adaptation should only be done if necessary	4	
Documentation (*n* = 70)^a^	Duplication with documentation in existing electronic medical record	24	☒
	Add the intended duration of treatment	3	
Adaption based on culture results (*n* = 63)^a^	There is often delay in the culture results	6	
	What to do if cultures are negative or not taken?	4	
	Not always feasible (immunocompromised patients, patients with cystic fibrosis, suspected joint prosthesis infection)	3	
Switch IV to oral treatment (*n* = 58)^a^	Item **3) ‘adequate in terms of diagnosis’ is not complete or too simplistic	18	☒
	It is not clear when and how to switch	6	
	This depends on the culture results	3	
General comments (*n* = 59)^a^	The checklist is not innovative	8	
	The item ‘antibiotic allergy’ is missing	8	
	Worries about extra administrative work for physicians	6	
	The need for careful implementation	5	
	The checklist is not feasible in all situations	4	
	Is this checklist evidence based?	3	

^a^Total number of comments on this checklist item

^b^Total number of participants who gave this comment

### Addressing identified barriers

We developed an implementation strategy that could be applied in all hospitals to address the top six anticipated barriers.

The barrier (1) “lack of expected quality improvement of the physician’s antibiotic prescribing” can be addressed in the following two ways: first by showing the room for improvement, i.e. giving feedback on their current antibiotic use based on a baseline measurement, and second by providing evidence for a reduction in the length of hospital stay for the patient with adequate antibiotic use [33]. This information can be given during a kick-off lecture at the departments that participate in the cluster-randomised trial on the implementation of the antibiotic checklist [32].

The barrier (2) “lack of expected patients’ satisfaction with checklist use” can be addressed by giving information about the study at the nursery department, i.e. by supplying flyers about antimicrobial resistance and the expected effects of appropriate antibiotic use [33].

Two anticipated barriers, namely (3) “the feasibility of the checklist” and (5) “the complexity of the checklist”, can be addressed by adapting the checklist. Table 4 shows the adapted checklist based on the survey results, which includes tick boxes and pre-printed options.

**Table 4:**
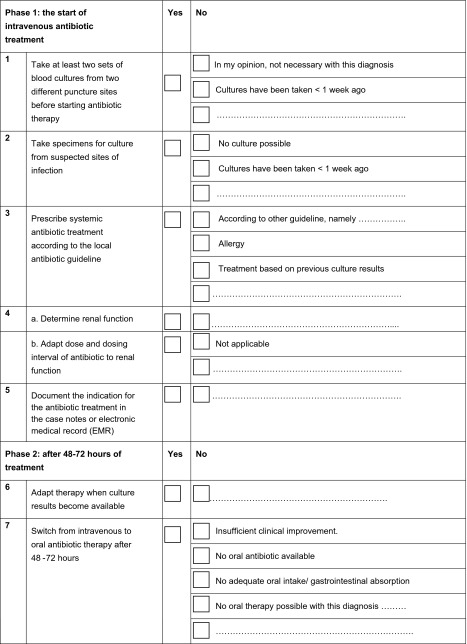
The final antibiotic checklist

To address the barrier (4) “negative previous experiences with other checklists”, the differences between these previous checklists (most probably the surgical safety checklist) and this antibiotic checklist should be emphasised, for example in group discussions. Differences are, e.g. that this checklist is short and does not involve other physicians to complete it. In addition, it should be stressed that the antibiotic checklist was adapted based on their comments and the comments of other colleagues participating in the trial.

Finally, to address the barrier (6) “lack of nurses’ expectation of checklist use”, nurses working at participating departments should be informed about the checklist use and its aims, for example through email, e-learnings and/or the department lecture showing the room for improvement and the evidence for reduction in the length of hospital stay for the patient with adequate antibiotic use.

### Incorporating comments

We adapted the checklist design based on the top five comments. First (1c), to reduce duplication of documentation, we left out the name, dose, interval and route of administration of the antibiotic, and the item was reduced to ‘documentation of indication’. This is appropriate since all Dutch hospitals are, nowadays, obliged to work with an electronic medical record that already requires this information. Second (2c), since 10.5 % of the participants felt that taking blood cultures was not necessary for all diagnoses, we added the option “In my opinion, not necessary with this diagnosis” in the checklist. Third (3c), we left out the clarification of “adequate in terms of diagnosis” for the item IV to oral switch, and included options in the checklist so that the physicians could tick their reasons for not switching: “Insufficient clinical improvement”; “No oral antibiotic available”; “No adequate oral intake/gastrointestinal absorption” and “No oral therapy possible with this diagnosis”. In addition, we ensured that the information on switch criteria could be easily found: we developed informative posters and laminated pocket versions of the checklist, which included a reference to the website with all necessary information concerning this topic. The information about dosage adaptation when the patient has an impaired renal function (4c) can also be found on this website. Last (5c), we added in the checklist that, for taking two different blood cultures, one should use two different sites.

## Discussion

In this study, we identified the barriers that need to be addressed when implementing an antibiotic checklist. We created a top six of anticipated barriers to the uptake of the checklist, and a top five of comments on its components. The top six barriers encompassed four different domains, namely barriers related to the individual professional, to the patient, to professional interactions and to the checklist itself. The top five comments mostly encompassed suggestions to more clearly specify the content of the checklist, i.e. the quality indicators included. We adapted the checklist to the survey results; two barriers and all top five comments could be addressed by adapting the design of the checklist, and by re-phrasing various checklist items (Table 4).

Our most frequently mentioned barrier, the lack of expected improvement of care, has been described by several other barrier studies of checklist implementation [17, 18, 20].

Feasibility is also a known barrier in the implementation of checklists; items in the surgical safety checklist were perceived to be inappropriate for certain surgical procedures [16] or for certain settings [18]. Patient perceptions were only mentioned as a barrier by Russ et al., describing that too many checks can create anxiety and unsafe feelings towards the system [16]. This might be an explanation of our anticipated barrier “lack of patient’s satisfaction”. However, this anticipated barrier can also be explained by the fact that our antibiotic checklist is typically meant as a reminder for physicians; in daily practice, patients probably will not notice the use of the checklist. Next, the literature describes barriers comparable to our sixth anticipated barrier, “lack of nurses’ expectation of checklist use”, since it is associated with both the lack of teamwork [20, 21, 23, 24] and professional hierarchy [17, 18]. Not surprisingly, the barrier “negative previous experiences with other checklists” was not mentioned in previous studies, as those evaluations were performed after the introduction of the checklists, and our inventory was performed before checklist implementation. The complexity of the checklist was once earlier described as a barrier after the implementation of a quality improvement checklist on an inpatient hepatology service [21]. It was, however, not a major barrier, as it was only mentioned by 1 of the 23 participants. The explanation for why this is a top five barrier in our survey and not in previous studies might be that other barrier studies have been done during or after checklist implementation. In that phase, the physicians’ main concerns are the organisational problems they are facing in daily practice and, consequently, these studies mainly describe logistic barriers, such as difficulties in timing (when to fill out the checklist) and lack of time [16–19, 21, 24], and professional barriers such as lack of senior support and professional hierarchy [17, 18, 20, 24]. We identified the barriers before implementation of the checklist, and the physicians, therefore, may have felt that they still had an influence on the contents and design of the checklist. Finally, it appears that the benefit of the use of checklists is not always clear [17–19] and a lack of understanding of the purpose of the checklist might influence its use [17]. This topic was, however, not mentioned as a barrier in our study.

The current study has some strengths. To our knowledge, our study is the first to assess and quantify barriers prior to checklist implementation. We based our questionnaire on a validated instrument to measure determinants of innovations, combined with a literature search. The barriers to the uptake of the antibiotic checklist were reported by a large sample of physicians from nine different hospitals. Furthermore, we included residents and specialists of surgical departments and medical departments, while previous barrier studies only focussed on people in the surgical [16, 18–20, 23, 24] or medical fields [15, 17, 21]. Finally, barrier identification prior to implementation resulted not only in the identification of concerns about the structure of the checklist and the introduction into the clinical workflow, but also concerns about the actual content of the checklist. Although the set of quality indicators has been developed in a RAND-modified Delphi procedure by experts [3], the comment “doubts about the need of blood cultures for several diagnoses” (2c) expresses disagreement among physicians about targeting “taking blood cultures” in the checklist. Disagreement about clinical measures targeted in the checklist can hinder implementation. Therefore, the option “no, in my opinion, not necessary with this diagnosis” was added in the checklist. The cluster-randomised trial [32] will show how often this option in the checklist will be used, and can help to determine whether taking blood cultures is part of agreed-upon standard concerning appropriate antibiotic use among frontline clinicians.

The most important limitation of our study is that the physicians of one university hospital completed 90 of the 219 questionnaires. We compared, however, the appraisals of the 219 participants (nine hospitals) with the appraisals of 129 participants (eight hospitals). This comparison resulted in the addition of a sixth anticipated barrier on nurses’ expectations regarding checklist use. During the implementation process, this barrier should also be addressed. A second limitation has to do with the chosen recruitment method. We emailed the contact physician a link to the questionnaire, and asked him/her to forward this email to the target group. Through this approach, we aimed to invite as many professionals as possible to participate in the questionnaire study. The downside of this approach is that the exact number of physicians that received the link to the questionnaire was unclear and, consequently, that it was impossible to determine the response rate. Based on an analysis in one hospital where we could retrieve information on the number of invitations sent, we estimated the response rate to be about 30 %, which is also found in other studies [34, 35]. Although the response rate is said to be important to determine the non-response bias, Willis et al. showed that increasing the response rate by additional re-contacts had little effect on the key data distribution and, therefore, they suggest in physician surveys to have a larger initial sample (as we did) and to accept a lower final overall response rate [36]. Furthermore, a barrier study prior to implementation also has its limits, as the appraisers lack experience in actually using the checklist. Finally, although the contents of our questionnaire were carefully chosen, some results might seem less informative after all. For the anticipated barrier concerning negative previous experiences, it remains unclear whether the problem with other checklists concerned, for example, the length of the checklist, the lack of time or no perceived benefit. Explicitly discussing this barrier in small groups of physicians—as suggested—might, however, provide insight into the underlying problem.

Performing a barrier analysis prior to checklist implementation creates the opportunity to address barriers in an early stage, and to adapt its design structure and content. This iterative survey resulted in a checklist adapted to the perceptions of frontline clinicians, which should facilitate implementation. The results of our barrier analysis may be specific for our setting and our checklist. Since our developed questionnaire is based, however, on a validated instrument and a literature search, we assume that the checklist questionnaire itself can be used for barrier identification for the implementation of other checklists.

This survey provides insight into the anticipated barriers that have to be addressed when implementing an antibiotic checklist in Dutch hospitals. Taking into account these factors that hinder the uptake of the antibiotic checklist, implementation in daily practice will be challenging. Education, feedback, involvement of the whole healthcare team in the implementation process and adaption of the checklist itself (Table 4) will be necessary to overcome the barriers hindering the uptake of the checklist and to improve the appropriate use of antibiotics. Our assumption that the suggested combination of interventions should facilitate successful checklist implementation will be tested in a cluster-randomised trial [32].
